# Alluvial fans at Cala Gonone (Sardinia), a fast developing touristic village: origins, hazards and potential risks

**DOI:** 10.1007/s12665-022-10638-9

**Published:** 2022-11-29

**Authors:** V. Pascucci, I. P. Martini, S. Andreucci

**Affiliations:** 1 Department of Architecture, Design and Planning, University of Sassari, Sassari, Italy; 2 School of Environmental Sciences, University of Guelph, Guelph, ON Canada; 3 Department of Chemical and Geological Sciences, University of Cagliari, Cagliari, Italy

**Keywords:** Climate change, Debris flows, Sheet flood, Glacial stadial, Late Quaternary

## Abstract

The study area of Cala Gonone in NE Sardinia (Italy) consists of a wide terraced re-entrance/valley crowned inland by carbonate hills and, near the coast bounded laterally and partly floored by thin basaltic lava lying over carbonate bedrock. In this re-entrance, several inland alluvial fans (500 m length by 700 m wide) have developed, and a local ~ 30 m high, about 10 m wide (thick), 400 m long scarp body-remnant of semi-consolidated alluvial fan deposits is exposed along the coast. The fans experience depositional events mostly developed during the late Pleistocene. They although nowadays dormant may be reactivated by major rainstorms during strong climate changes. In these last few decades, the touristic village of Cala Gonone has been rapidly expanding over the mid to lower parts of two coalescing alluvial fans (Stadium and Gustui) and along the coastal marine scarp edge (Palmasera alluvial fan system). The village thus may become exposed to natural hazards if extreme climatic conditions may re-occur. Moreover, rock falls and the instability of the costal scarp due to wave erosion may add addition hazards for habitations built near the scarp crest and visitors to the frontal replenished beach. As commonly occurring elsewhere since antiquity, the risk perception of such events is low because of the centennial, millennial of longer recurrence. Such perception does not negate the hazards but a long event recurrence may be accepted as a reasonable risk for the human’s activity. Nevertheless, serious consideration should be given to potential problems and plan and build for amelioration and defense. The evidence of what environmentally did and could still happen in the Cala Gonone and similar other area is in part clearly imprinted on the landscape: geology, geomorphology, and relative details in the stratigraphy and sedimentology of the deposits.

## Introduction

Modern concerns/applications of Sedimentology are increasingly related to environmental problems. Accordingly, the approach of this study has been to examine some specific environments, such as the alluvial fans, not just as isolated sedimentary bodies (their sedimentological characteristics and infer the processes involved), but also as part of the entire (natural and cultural) landscape (geology, geomorphology, climate, hydrology and natural hazards) (Clemmensen et al. 2001; Martini and Chesworth, 2010).

Alluvial fans are depositional features, formed of variety of sediments from clay to coarse gravel transported by mass flows such as debris flows and or heavily sediment-loaded floods. The resultant landforms are usually fan-shaped in plane and wedge-shaped in profile (Bull 1977; Blair and McPherson 1994, 2009; Harvey et al. 2005; Clark 2015; Bowman 2019). They occur commonly in two topographic situations: at mountain fronts and at tributary junctions (Harvey 1997). Fans range in axial length up to 10 s of km, though many fans described in the literature range in size between ca. 100 m and a few km (Harvey et al. 2005). They can form under most variable climatic conditions and change characteristic from debris flow to alluvial flow dominated and composite ones. As scale increases, there is a tendency for the dominant process to change, from small debris-flow dominated tributary-junction fans (debris cones), to mixed processes and flood dominated at intermediate scales, and by channelized fluvial flows at the largest scales (Harvey 1997). Alluvial fans preserve a sedimentary record of environmental change, and act as major controls on the downstream fluvial system, often breaking the coupling between sediment source areas and distal fluvial environments (Harvey 1997).

Alluvial fans have been used for habitations, other constructions and cultivations since antique times in most populated area of the planet. Roman villas, for example, were located three quarters to halfway the slope to have an upper grazing and a cultivated terrain below, above the unhealthy, fog-ridden valley-plane lands (Martini and Wightman 1987). Some ancient inhabitants, such as the Bronze Age and Nuraghi in Sardinia utilized the gentle fan slopes, but built their large villages on the fans flank on solid-land not subjected to floods and mass-flows (De Palma and Melis 2010). During other times, societies, such as the Iron-Age people, for safety reason avoided the gentle slopes and plane areas (Cesaretti, 2012). However, returns have occurred up to the recent times to the usage of fans also for permanent settlements, although natural hazards such as slumps, debris flow, floods of various types and the inevitable resulting risks were and are well-understood. Extensive remediation has been done in some cases, in others the long recurrence of disastrous events does not require numerous and expensive defences (Ryzner and Owczarek 2020; Walstra et al. 2010; Ferring 2001).

The processes involved, benefits and hazards have been extensively studied and reported (Antronico et al. 2016). Commonly the processes have been directly observed during or soon after the events including reporting the damages that may have occurred, or through experiments (Arattano et al. 2010; Antronico et al. 2016).

**Fig. 1:**
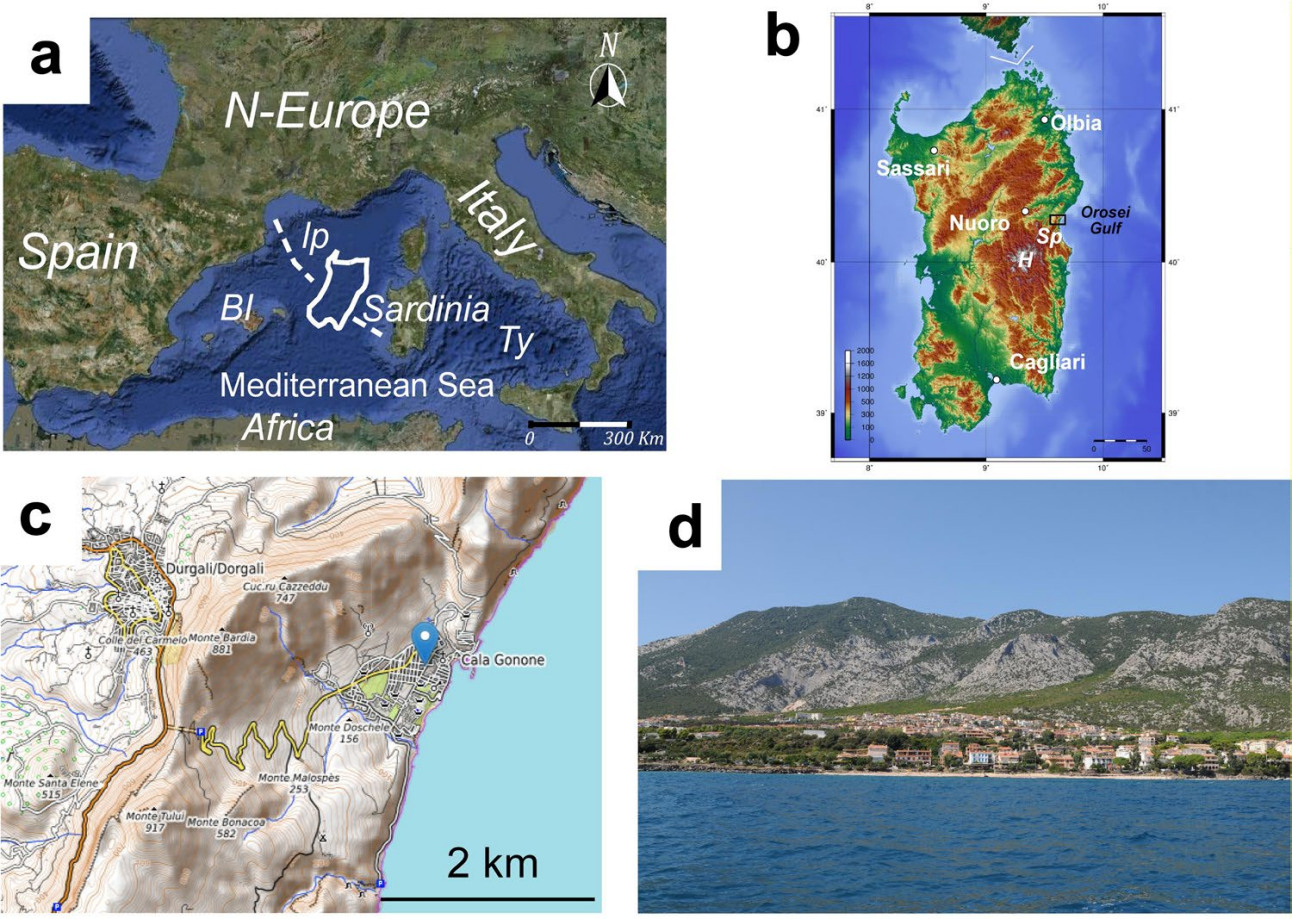
Study location at Cala Gonone, northeastern Sardinia. **a** Satellite view of the Mediterranean region where Sardinia occupies a central position. Dashed line indicates the Sardinia anticlockwise rotation occurred in the Neogene time. Abbreviations: BI = Balearic Islands; lp = Liguro-Provençal Basin; Ty = Tyrrhenian Sea.; **b** Digital elevation model of Sardinia; in the map are reported the main cities. With ***Sp*** is indicated the area close to Cala Gonone (locally known with the name of Supramonte of Dorgali), with ***H*** the intermontane basins and high mountains (up to 1800 m) in the centre of the island. In the square the studied area of Cala Gonone. The image can be freely downloaded at: https://commons.wikimedia.org/wiki/File:Sardinia_topo.png#file) (Image details: horizontal resolution = 118.11 dpc, Vertical resolution = 118.11 dpc, File change date and time 11:03, 20 November 2012; **c** Cala Gonone (belonging to the municipality of Dorgali) is located on a coastal terrace at the base of steep mountain flanks (Image from road map of Google Map). **d**) Sea view of the village of Cala Gonone built on alluvial fans (photo courtesy of Augusto Perez Alberti)

**Fig. 2:**
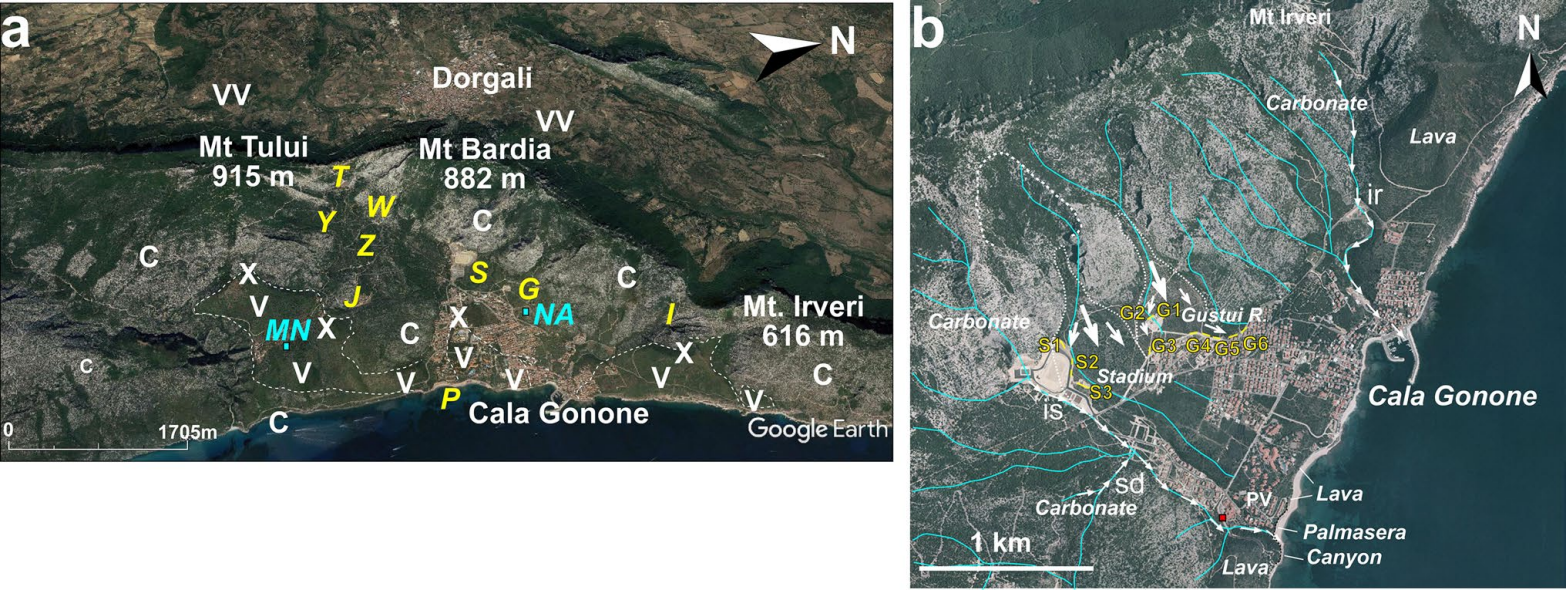
Cala Gonone area. **a, b** Major morphological features. The white dashed line indicates the boundaries of lava flows; **b** detail of the Cala Gonone surrounding area where are reported the streams (blue lines), the Stadium (**S**) and Gustui (**G**) alluvial fans, and the Rio Sos Dorroles(**sd**) and codula corridor leading to the coastal alluvial fan Palmasera (**P**). Abbreviations: **C**: Mesozoic carbonates; **V**: uppermost Plio-Pleistocene basalts; **VV**: Palaeozoic-to-Pleistocene volcanics; **X**: Plio-Pleistocene volcanic vents; **I**: Irveri valley; **T:** Bardia saddle; ***S***: Stadium alluvial fan;*** G***: Gustui alluvial fan;* Y,W, ****Z****, ****J***: outcrops of residual parts of talus cone deposits in the inland higher valley of Cala Gonone mostly visible along the road SP26 ; **P**: coastal Palmasera alluvial fan system; **MN:** Nuraghe Mannu; ***NA***: Nuraghe Arvo; **ir**: Rio Irveri; **sd**: Rio Sos Dorroles Torrent and codula; **is**: Rio Ischirtiore. The red square indicates the position of buildings of Fig. 7h. (Images from Google Earth).

In this paper, we are not reporting on a damaging event, rather on the information that pre-existence given that sedimentary deposits can provide to guard and prepare for future inevitable damaging occurrence albeit uncertain on its recurrence time and intensity. The objective of this paper is, thus, to contribute to recognize the potential natural hazards and risks through the study of the Pleistocene sedimentary deposits of an alluvial fan systems fed from steep highlands and expanded across elevated and cliffed shores in Sardinia Island (Italy) (Fig. 1a, b). This deals with a coastal area of the Gulf of Orosei, Cala Gonone, in east Sardinia (Fig. 1b, c). Cala Gonone historically became a small harbour for Italian fisherman at the beginning of 20^th^ Century but remained isolated from the rest of Sardinia because of the impervious high costal hill barrier (Fig. 1c, d). In 1860 a small walking tunnel for people and animals was perforated near the saddle between the Mt Tuli and Mt Bardia (Fig. 1c). As the village prospered, particularly for the recent touristic rapid increase, modern motor-vehicle tunnels were open along the road to Dorgali (main centre nearby; Fig. 1c) on the northwest flank of the coastal hill barrier (Figs. 1c, 2a). The touristic village of Cala Gonone has been rapidly expanding on these last few decades over the mid to lower parts of alluvial fans and along the coastal marine scarp edge (Figs. 1d, 2). It is now a preferred touristic area for good beaches and various activities such as unusual canyoning, climbing, paragliding, and all other activities offered, receiving pleasant touristic hospitability. More than 600.000 tourists per year enjoy the place with a constant increase of presence of about 15% per year (data before COVID 19 pandemia from SIRED, http://osservatorio.sardegnaturismo.it).

The specific study area of Cala Gonone consists of a triangular shaped wide re-entrant valley crowned inland by carbonate hills (Mt Tului, Mt Bardia, Mt Irveri) (Fig. 2a), and a gently seaward-dipping carbonate terrace partly covered by thin basaltic lava flow (Fig. 2a). In this re-entrance, several varied coalescent talus cones (***T,Y,W****, ****Z****, ****J***) and alluvial fans (inland **S**: Stadium, **G**: Gustui; and the coastal **P**: Palmasera, Figs. 1d, 2) have developed.

The geological (rock source of the sediments and morphology of the terrains allowing for their movement and space for deposition), meteorological (temperate, wet and dry conditions establishing the regular or variable precipitations of rain and snow), hydrologic distribution and anthropogenic activities (conform or not to natural hazards) establish the existing landscape (Fig. 1d).

### Geology

**Fig. 3:**
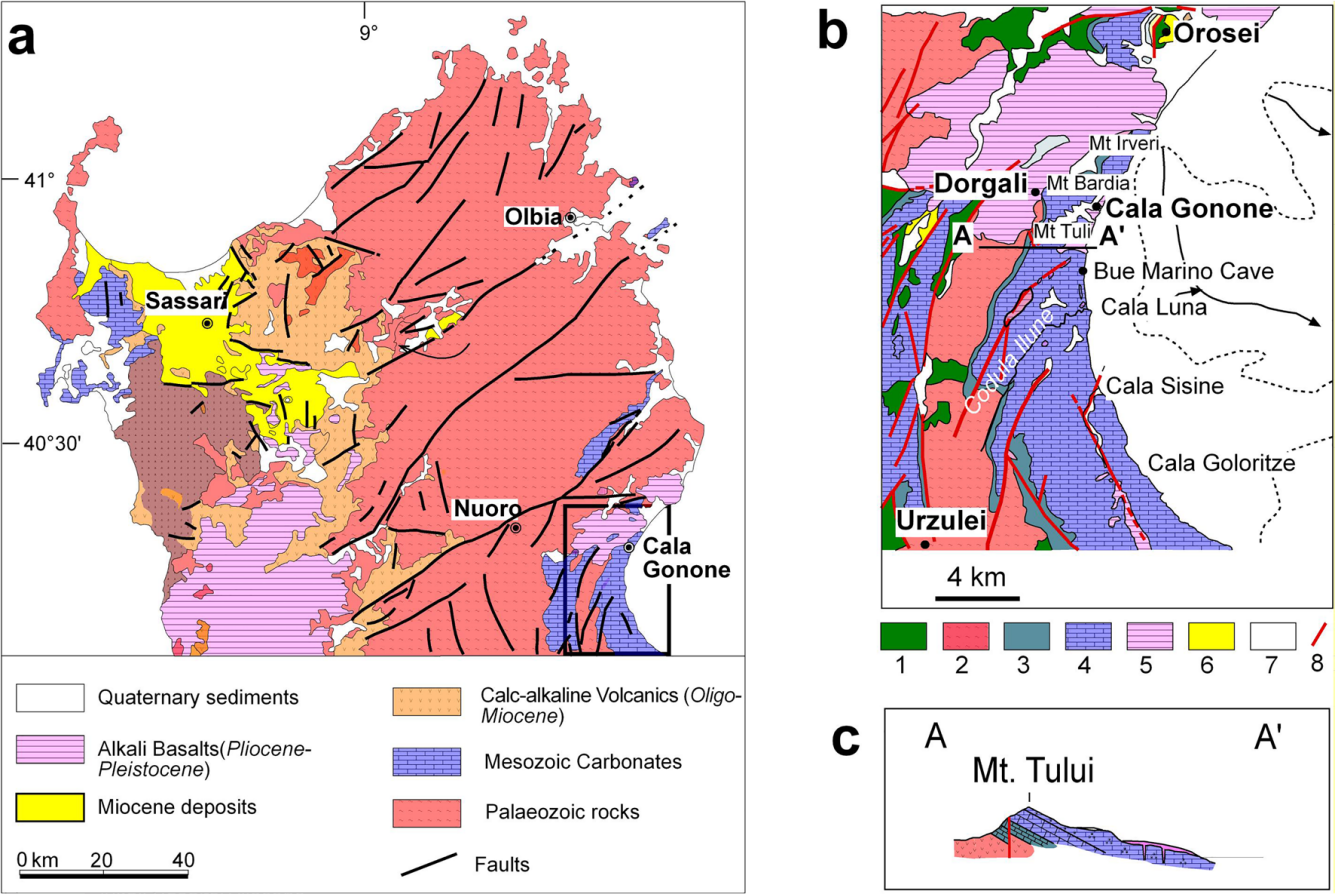
Geology of north Sardinia. **a** Simplified geological map of the north-central part of Sardinia; **b** geological map of the Cala Gonone area: 1—Palaeozoic metamorphic rocks; 2—Late Palaeozoic granites; 3—Jurassic dolostones; 4—Jurassic–Cretaceous limestones; 5—Plio-Pleistocene basalts; 6—Plio-Pleistocene sedimentary deposits; 7—late Pleistocene–Holocene deposits; 8—faults; dashed line indicate -50 m water depth, solid lines with arrows the two main marine canyons developing in front of Cala Gonone and Orosei (after New Geological map of Italy 1:50,000 scale—Foglio Nuoro Est, available at: https://www.isprambiente.gov.it/Media/carg/500_NUORO_EST/Foglio.html); **c**) Cross section of the Mt. **c)** Bedrock dipping eastward structure of Mt. Tului (location is in Fig. 3b),(after Lanfranchi et al. 2011)

The island of Sardinia detached from the European plate during early Miocene and drifted eastward to the late Miocene when it reached its present position in the Western Mediterranean Sea. Presently it constitutes the eastern continental margin of the Balearic Sea and western margin of the Tyrrhenian Sea (Cherchi and Montardert 1982) (Fig. 1a). Both sea basins have been affected by rifting to drifting processes that eventually led to the formation of an oceanic crust in their central deeper part (Rehault et al. 1984; Sartori et al. 2004; Marani et al. 2004). Normal and transcurrent faults dissected Sardinian pre-Miocene deposits and allowed the formation of a series of half graben that were filled with Miocene continental and marine deposits and calc–alkaline andesitic lava flows (Carmignani et al. 2001; Casula et al. 2001; Gaullier et al. 2009) (Fig. 3a). During the Pliocene widespread alkali basalt volcanism and basins uplift was followed by local renewed dissection and tilting of bedrock blocks (Casula et al. 2001; Funedda et al. 2000) (Fig. 3a). Since the Early Pleistocene Sardinia is considered tectonically stable (or quasi stable) (Cocco et al. 2018; Casini et al. 2019), and the various marine to continental sedimentary successions are the result of repeated variations of Quaternary global climatic changes (Fig. 3a) (Andreucci et al. 2009; Pascucci et al. 2014, 2018). These led to the formation of well-developed costal systems formed by a transgressive sequence passing upward from shallow marine to beach/dune to alluvial deposits (Andreucci et al. 2014; Pascucci et al. 2014; Sechi et al. 2020; De Falco et al. 2022).

The present geological setting inland from the Gulf of Orosei consists of two Mesozoic (Jurassic to Cretaceous) sub-parallel N–S-oriented carbonate ridges separated by a Palaeozoic granitic–metamorphic zone in part covered by Pliocene basaltic lava flows (Beccaluva et al. 1985; Carmignani et al 2015) (Fig. 3b). The costal ridge mainly consists of seaward steeply dipping (20–30°) layers composed of Jurassic carbonates (Jadoul et al. 2010; Lanfranchi et al. 2011) (Fig. 3c). The coastal ridge is cut by several NE–SW transcurrent and variably oriented normal faults along which few small volcanic vents have ejected lava thinly mantling some coastal areas approximately 1.99–2.83 Ma ago (Fig. 3b, c) (Dieni and Massari 1966; Massari and Dieni 1973; Savelli and Pasini 1973; Beccaluva et al. 1985; Carmignani et al. 2015).

The carbonates coastal cliffs of the Gulf of Orosei have numerous karstic caves and are dissected by deep, narrow canyons (“codule” in Sardinia) with ephemeral streams flooding on decals or longer intervals (Fig. 3b) (Cossu et al. 2007a, b; De Waele 2004; De Waele et al. 2010). Only one of these streams (Codula Ilume) has its headwaters in the granitic/metamorphic inland zone, cuts through the coastal carbonate range and carry some metamorphic rock to the coast at Cala Luna, occasionally remoulded by large codula floods having a recurrence of about 130 years (Cossu et al. 2007a, b) (Fig. 3b).

Several colluvial and alluvial fans have developed at the base of the coastal cliff of the Gulf of Orosei, among which the alluvial Palamasera (**P**) fan at Cala Gonone (Martini et al. 2018). Tidal notches, locally well-developed, mark the lower part of the cliffs from ~ 7 m up to 10 m above the present sea level recording several sea high stands induced by global Pleistocene climatic changes (interglacials) (Carobene 1972; Antonioli et al. 1999).

Major canyons down to deep sea floors characterize the submarine area. The canyons developed during the low stand of the Messinian salinity crisis of the Mediterranean Sea (Hsu et al. 1973; Ryan 2009) and were partially modified during the low sea-level stands of the Pleistocene glacial periods (Giresse et al. 2014) (Fig. 3b).

### Climate

Sardinia has now a typically Mediterranean temperate climate tending to warming-up. It is characterized by very dry summer months, cool winters with annual snow fall in mountain over 1000 m above sea level (asl) (***H*** of Fig. 1b) and frequent occurrence on hills around 500 m as l (Canu et al. 2014). During wet seasons (fall and early winter) it experiences local extreme rainstorm events with sudden downpours of 431 mm in few hours particularly in central and northeastern regions (south of Nuoro, Fig. 1b) (Cossu et al. 2007a, b, 2010; Betrò et al. 2010; Bodini and Cossu 2010a, 2010b; Hewson et al. 2021). These strong precipitations, occurring during the fall in eastern Sardinia, are related to the Atlantic cyclonic flow that crosses North Africa and the warm southern Mediterranean Sea (Nuissier et al. 2008; Ricard et al. 2012) impinging on the Sardinia highlands (Pensieri et al. 2018). Particularly affected are the intermountain basins of the eastern highlands (between ***H*** and ***Sp*** of Fig. 1b) but occasional downpours occur also along the coastal mountains and hills such as those of the Cala Gonone area (***Sp*** of Fig. 1b), being also exposed to the northern and eastern wind induced storms. The marine storms produce almost yearly strong high waves (order of 7 m) disruptive of the coastlines (ISPRA 2004).

Cala Gonone had colder conditions during glacial times, with small glaciers occurring in higher about 1000 m asl inland areas of Sardinia and more in the adjacent Corsica Island (Hughes and Woodward 2016). The frigid glacial-time temperatures led to frost cracking generating large quantities of carbonate clasts, continued to be formed in minor measure during the recent winter ground-frost and fewer freeze-and-thaw cycles.

## Methods of study

Available multidisciplinary information has been reviewed and new obtained dealing with the geology, sedimentology, geomorphology, and climate of the area, and with mass-wasting and flood processes. The major fans have been defined in term of sedimentary features using lithofacies analysis. The most commonly occurring facies (aspects) have been described and photographically documented (Table 1). Where possible, major lithostratigraphic units have been defined utilizing differences in lithofacies assemblages and separated by key horizons.

**Table 1:** Principal processes and resulting sedimentary facies of the Quaternary deposits of ‘Cala Gonone’ fans

.Name Transport mode	Label	Lithology/texture/ colour	Structure	Location	Image
Rockfall saltation, rolling; minor matrix	rf	Carbonates/ angular boulders to pebbles, minor sand to finer- grained matrix; poorly sorted	Isolated clasts to disorderly remobilized, massive clast accumulations	Steep slopes and basal boulder at cliff foots	
Grain flow Sediment – gravity flow	gf	Carbonate angular pebble to coarse sand, moderate sorting, generally open framework and ~showing inverse grading	Lensing beds	Preferably in steep slopes and local deposit inhomogen eity	
Debris flow noncohesive Mass wasting	dg	Carbonate boulders to angular cobbles, pebbles to sand, poor sorting	Massive thinning out gravel beds	Steep to moderately inclined slopes	
Debris flow cohesive Mass wasting and flow	df	Carbonate angular boulders to cobble and finer clasts, abundant silty to clay (mud) matrix	Massive, to thinning out strata, matrix- supported deposit.	Steep slopes and spreading onto lower terrains	
Sheetwash / Sheetflood (overland traction flows) with various sedimentary loads. To: Flashflood carrying in and reworking material on fans	sh/sf	Carbonate sand to angular granules to small cobbles, moderate sorting Variable carbonate clasts with sandy to muddy matrix	Thin beds, laminated, thinning out (in part due to erosion} Hin to tick layers grading to thinner strata downflow	Variable slopes of alluvial fans and lower terrains Variable slopes	
Channel flow Sediment transported and deposited by canalized flow	cf	Carbonates, subangular to surrounded pebbles to cobbles, sandy matrix, moderate sorting, frequented regular grading,	Plane to cross- bedded, local massive, thinning out. Presence of cut-and-fill structures	Moderate slopes of alluvial fan and flatter alluvial plains.	

No numerical ages have been obtained directly from the alluvial fan deposits because mostly made of carbonate and minor basalt clasts. However, the analysis of organic matter obtained from a presumed basal paleosol layer of the coastal Palmasera fan provides radiocarbon ages (^14^C) of 19,365–17,485 y BP (Coltorti et al. 2010; Thiel et al. 2010). Quartz and K-feldspar grains obtained from approximately the same horizon in a different place along the geological cross section were dated using both OSL (optically stimulated on quartz) and post IR IRSL (post infrared stimulated on K-feldspar) luminescence methods. Samples were prepared under controlled red light conditions following the conventional procedure to obtain pure quartz and K-feldspar grains. Luminescence analyses were conducted at Luminescence Laboratory of the Sassari University. Measurements were made using two automated Risø TL/OSL readers (DA-20 and DA-15; Bøtter-Jensen et al. 2010) with calibrated 90Sr/90Y beta sources (~ 0.15 Gy/s and ~ 0.08 Gy/s). The OSL age of quartz samples is limited by the saturation of the natural signal. Therefore, K-feldspar SAR post infrared stimulation (post-IR IRSL) at elevate temperature (290 °C) protocol was applied to the samples for which an enough grains were obtained (Thiel et al. 2010; Andreucci et al. 2017). A date of 87 ± 4 ka was obtained.

Other upper Pleistocene dates are also available from literature for rocks associated with the base of the Palmasera cliff. The K–Ar for the Basaltic lavas of the area provided a numerical age of about 2.11 ± 0.15–2.54 ± 0.11 (Savelli and Pasini 1973) and 2.34 ± 0.05 (Sarria et al. 2016). Others were obtained from coastal deposits trapped inside the Bue Marino cave close by to Cala Gonone (Fig. 3b). Ages derived using both luminescence and U/Th methods range from 134 ± 32 to 86 ± 13 ka. The uppermost dated colluvium inside the cave has a luminescence (post IR IRSL) age of 86 ± 4 ka (Andreucci et al. 2017).

According to the above-mentioned points, manuscript will follow the interpretation of the sedimentary characteristic of the alluvial systems, the identification of their origin, their depositional pattern, the sediment source, transport and chronology. Finally, the potential hazards and risks for the village of Cala Gonone will be analyzed.

## Results

The Cala Gonone fan system is made of a series of smaller telescopic and coalescent talus cones and alluvial fans developed along the inner valley from the saddle between Mt Tuli and Mt Bardia to Cala Gonone village at locations **T, Y, W, Z** (Fig. 2a). Lower fully developed alluvial fans affecting the village and costal area were/are fed by streams from steep coastal hills (Fig. 2b). Two well-defined and well-exposed inland ones are the Stadium (**S**) and Gustui (**G**) alluvial fans, which also contributed to the formation of the larger Palmasera coastal alluvial fan system (**P**) (Fig. 2). Minor sediment contribution to the apron system is also from northern Mt Irveri valley (Fig. 2b). The entire system is crossed by the ephemeral, steep mountain torrents the main ones being the Rio Irveri (ir), Rio Ischirtiore (is) Rio Sos Dorroles (sd) (Fig. 2b).

The fan system of Cala Gonone can be subdivided in three parts.

**Fig. 4:**
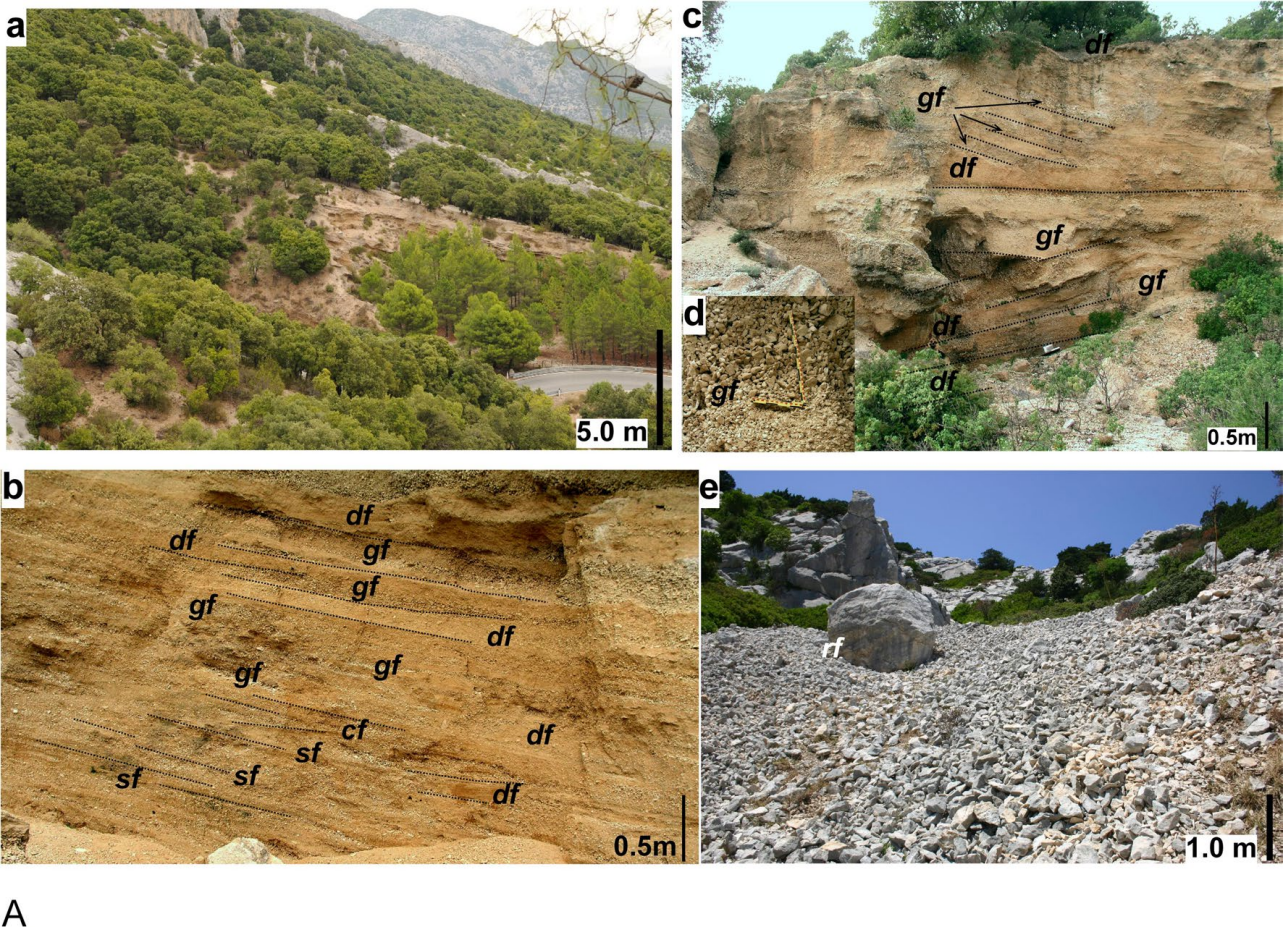
Deposits of the upper fan system-talus cone between Mt Tuli and Mt Bardia. **a** General view of the well stratified, steeply dipping deposits in the foreground, dipping Jurassic carbonate layers in the background; **b** typical internal stratification of reworked tunnelling clasts with indication of principal facies (*df*: debris flow to hyperconcentrated flow, *gf*: grain flow, *sf*: sheetflood/flash flood), **c** gully filled primarily with mass flow deposits (*df*: debris flow, *gf*: grain flow); **d** detail of grain flow layers, note the openwork framework and the inverse (coarsening upward) grading; **e** rock fall (rf) accumulation composed of heterometric, angular carbonate cobbles and pebbles with some isolated boulders (higher slope talus cones, Mt Irveri)

The uppermost part of the system develops in the valley between Mt Tului (915 m) and Mt Bardia (882 m). It has a length of approximately 500 m and width of 500–700 m (Fig. 2a). lt is made of slope deposits occurring in several lateral, small, steep-sided head-valleys developed along the mountain flanks (Fig. 4a). Locally they consist of lenses of greyish orange (10YR 7/4) to moderate yellowish brown (10YR 5/4), fine carbonate pebble to granule conglomerates (breccias) (Fig. 4b, c). They generally occur in openwork framework, frequently showing inverse (coarsening upward) grading (Fig. 4d) alternating with thinner lenses of poorly sorted, matrix rich (primarily sand with minor silt, and traces of dust as coating) pebbly to granule deposits, and few, thin, residual lenses of laminated coarse-grained sandstone (Fig. 4b, c). Occasional isolated boulders to cobbles are present (Fig. 4e). The layering consists primarily of thin elongated lenses dipping locally up to 30–32°, generally 20–25°. Small cut-and-fill structures are locally present (Fig. 4b); rare large gullies occur filled with a variety of slope deposits (Fig. 4c).

Near the apex, significant unconformities (diverging attitude of strata, or locally large gullies filed by mass flow deposits) indicate switching of sites of deposition (Fig. 4c). Non-cohesive and cohesive debrisflow (*df*), grain flow (*gf*), overland sheet-wash/flood (*sf*) and canalized flow (*cf)* are the recurrent depositional factors (Fig. 4b–d). In the saddle west of Mt Irveri closer to the steep calcareous hill, bouldery rockfalls (*rf*) prevail (Fig. 4e).

The alternation in these talus cones of openwork and sandy matrix-rich lenses could be explained as dry grain flows, perhaps forming during dry seasons/periods, alternating with small debris flows triggered by intense rainstorms during wet periods (Harris and Prick 2000; van Steijn and Hétu 1997; van Steijn 2011).

The lower, central part of the Cala Gonone system includes the Stadium (**S**) and Gustui (**G**) alluvial fans (Fig. 2). They have free-surface length of approximately 500 m and width of 500–700 m, and variable slope ranging from 20° at the apex to 2° in the low parts. They mostly consist of poorly cemented carbonate pebble, with some cobbles and rare small size boulders and variable amounts of sandy, muddy matrix. The clasts are generally angular to subangular. The sorting varies from poor to moderately well-sorted in different layers, which indicates variable transport agents. Both fans are now covered in the upper part by bushy vegetation, and the lower part is progressively anthropized by the enlarging village of Cala Gonone (Figs. 1d, 2b).

### Stadium alluvial fan (S)

**Fig. 5:**
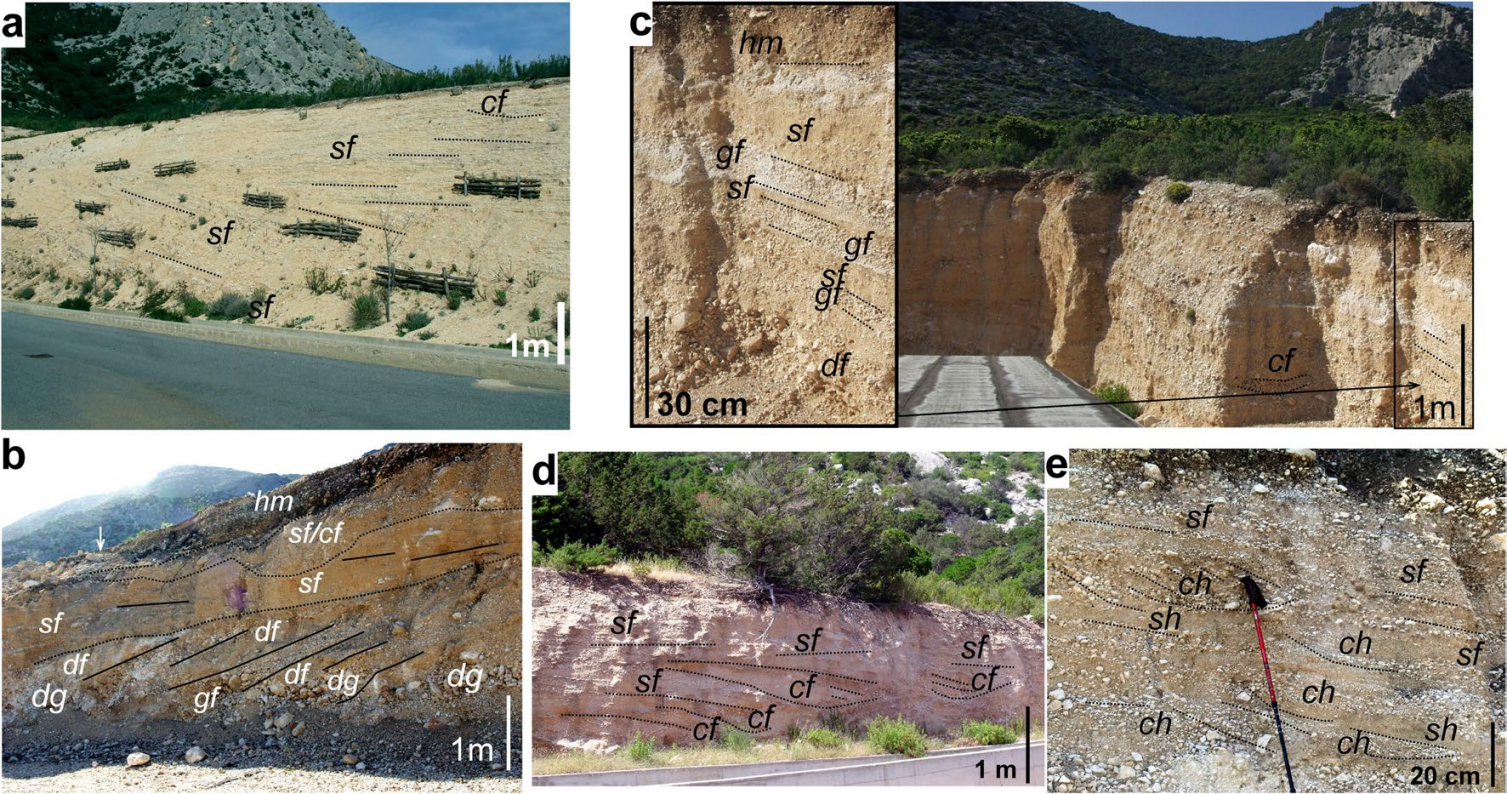
Deposits of the Stadium (**S**) and Gustui (**G**) alluvial fans. **a** Quasi-longitudinal section of the Stadium alluvial fan near the fan apex shows two superimposed systems with different dips mostly characterized by sheetflood/flashflood (*sf*) deposits; **b** general view of a deep-gully/flank of the Gustui fan showing three units; (i) a basal one dominated by debris flows (*df, dg*), (ii) an intermediate one dominated by sheetflood/flash flood (*sf*), and (*iii*) top one with sheetflood/flash flood (*sf*) and channel flow (*cf*) but heavily modified by man (*hm*). Everything is capped by modern soil. **c** Shallow longitudinal and transversal sections of **G** characterized by a quasi-rhythmic deposits of sandy, fine pebble conglomerate alternating (*gf*) with coarse grained sandstone to sandy granule conglomerate locally laminated (*sf*); **d** transverse outcrop in middle part of the Gustui Fan. North-eastern tail end of the alluvial fan deposits reworked by sheetflood/flashflood (*sf*) and channel (*cf*) floods overlain by rock debris fallen from the near carbonate bedrock; **e** alternation of sheetflood (*sf*) and channel flows (*cf*) sandy gravel deposits. Walking pole for scale (1.2 m)

The sediments of the Stadium alluvial fan are exposed in two long outcrop sections cut sub-longitudinally **(S1, S2** locations) and diagonally (**S3**) to the fan slope (Fig. 2). Closer to the hills the longitudinal section **S**1 shows two uncomfortably superimposed units with slightly different sediment characteristics and dip of the beds (Fig. 5a). The lower unit consists of steeper (~ 10–12°) thin, lenticular beds of very poorly sorted, sandy, muddy pebble to locally cobble, alternating with lenses of sand to granule deposits with disseminated fine grained pebbles and faintly lamination (Fig. 5a). The upper unit consists of gently sloping (~ 2–3^o^ in this exposure) thin, lenticular, muddy, sandy pebble layers alternating with thin sandier lenses and drapes (Fig. 5a).

The lower units were affected mostly by overland fluid flows, sheetflood/flashflood (*sf*) and, possibly, watery debris flows and channel flow (*cf*). The upper unit shows sandy conglomeratic horizon likely deposited primarily by sheetflood/flashflood (*sf*) and channel floods (*cf*) (Fig. 5a).

### Gustui alluvial fan (G)

The deposits of the Gustui alluvial fan are exposed in sand pits in its middle-upper part (**G1, G2**) and more extensively along quasi-transversal and diagonal road cuts in its downslope part (**G3 to G6**) (Fig. 2b). At **G2** locality three major stratigraphic units may be identified (Fig. 5b): (i) the lower unit consists of basal steeply inclined thick lenses of small boulder, cobble to pebble openwork conglomerate overlain by massive pebbly sand–granule deposits and very coarse sands with disseminated pebbles lenses. (ii) This is overlain by continuous, at the outcrop level, unit with apparently massive to slightly laminated sandy to granule deposit some with sparse pebbles, enclosing some pebbly lenses and small cut-and-fill structures. This second unit is cut in the up-dip part by a small channel filled with pebble to cobble openwork conglomerate. (iii) Everything is unconformably overlain by massive, disorganized pebbly sand-to-granule deposits with sparse pebbles and rare boulders, capped by organic rich, dark colored soil of similar lithology.

The entire deposit at **G2** records the infilling of a deep gully by powerful events. The lower unit is dominated by granular not-cohesive debris flow (the coarser lenses) (*dg*) and sandy/muddy matrix rich watery debris flows (*df*). The overlaying unit is dominated by sheetflood/flashflood (*sf*) possibly reworking also debris flows, and local channel flow (*cf*). The capping unit resembles the underlying one, being, however, partially reworked by man (*hm*). A thin, dark-brown, sandy pebbly soil caps the unit (Fig. 5b). No ancient soil horizon has been encountered in these sections.

An adjacent (20 m apart) thinner exposure at **G1** location a thin (~ 3 m) near top-fan surface exposure shows a regular alternation of clast-supported, fine pebble to granule conglomerate lenses resting with sharp bases on predominantly sandy layers with disseminated fine pebbles, and very fine pebble to granule laminas (Fig. 5c). This unit is best interpreted as an alternate of grain flow (*gf*) and sheetflood/flashflood deposit (*sf*) with some very small cut-and-fill structures (*cf*). A thin sandy pebble to cobble medium-thick conglomerate lens occurs at the base of this section possibly associated with a debris flow (*df*) event (Fig. 5c).

The transversal, middle, roadside exposures of both the Stadium and Gustui alluvial fans (**G4**, **G5**, **G6**, Fig. 2b) shows sandy pebble conglomerates occurring in sequences predominantly of cuts-and-fills channel flow and quasi regular alternation of thin lenses of sandy, pebble conglomerates, and granular very coarse-grained sandstones with sparse fine pebbles (Fig. 5d). These deposits here and along the road outcrops are interpreted as formed by debris flows reworked in part by sheetflood/flashflood (*sf*) and shallow channel flows (*cf*), possibly in a braided system (Fig. 5d, e). Occurrence of local unsorted sandy pebbles to pebbly sand deposits suggest the presence of mid to distal parts of debris flow (Fig. 5e).

Rock-falls of carbonate blocks from a nearby cliff occur just off the northeast lateral terminal part of the Gustui fan (Fig. 2b). This indicates that tail ends of debris flows, muddy sandy gravely floods (and local and minor rockfalls) did reach the extreme edge of the present village and possibly could also reach the entire area down to the coast.

### Coastal Palmasera alluvial fan (P) (lowest part of the system)

**Fig. 6:**
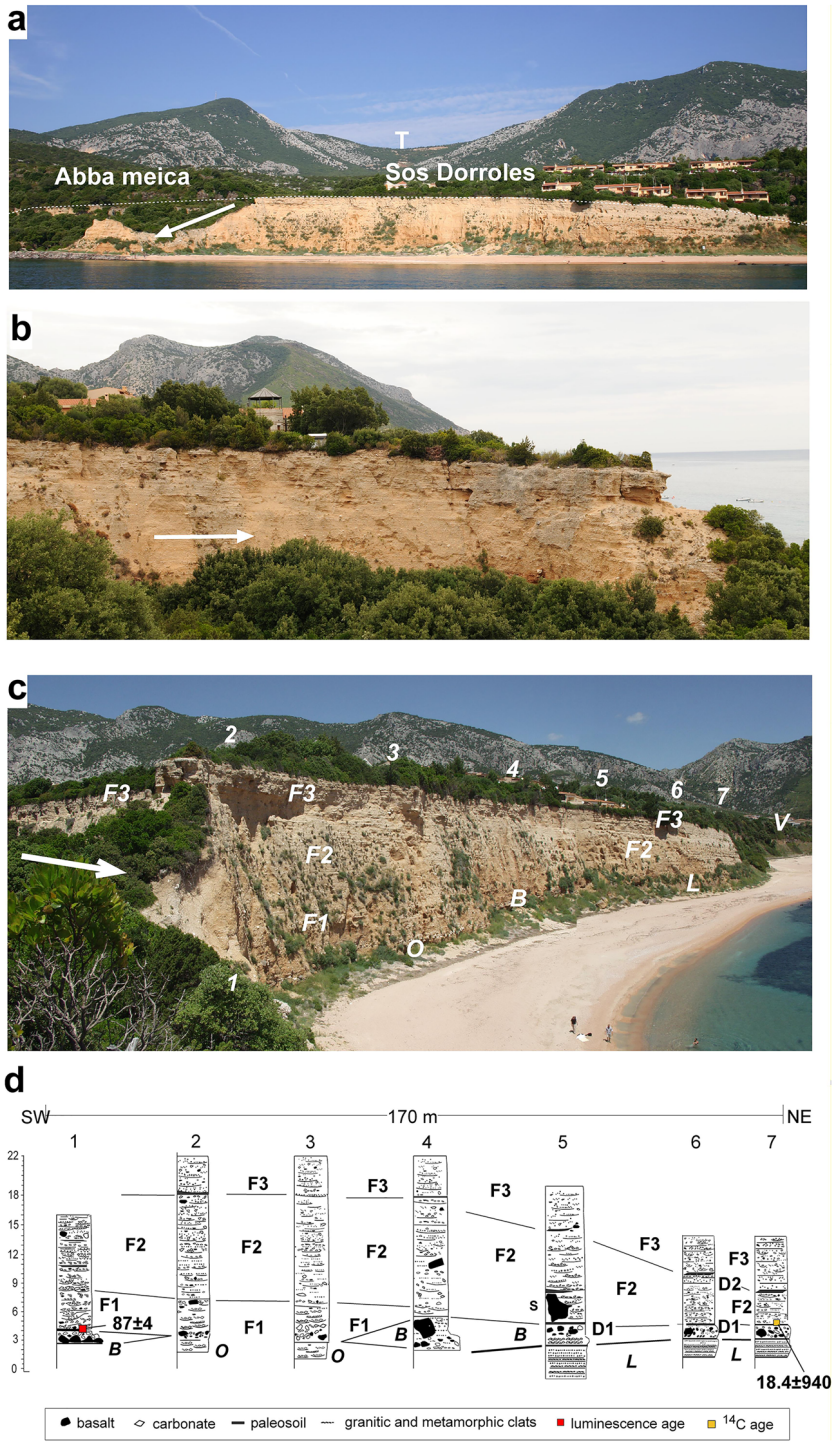
Sedimentary deposit of coastal alluvial Palmasera fan close to the village of Cala Gonone. **a** Transversal view of the Palmasera alluvial fan complex. **T**: saddle between Mt Tului and Mt Bardia at the top of the longest valley from Cala Gonone. Position of Abba Meica and Sos Dorroles beaches. These two beaches were renurished for touristic purposes and to prevent the erosion of the Palmasera cliff in 1990 (Pranzini and Mania 1996). Arrow indicates the condition of the northeastern part of the modern Sos Dorroles codula corridor; **b** transversal view of the Palmasera alluvial fan complex along the left flank of the codula. Arrow indicates the flow direction from the codula. **c** Subdivisions into lithostratigraphic units of the Palmasera fan along the Sos Dorroles beach (arrow indicates the direction of the flow of the Sos Dorroles codula). 1–7 stratigraphic logs location. **d** Schematic logs (1–7) of stratigraphic sections of the exposed fan from the codula mouth (1) and along the Sos Dorroles beach with tentative litho-subdivisions (2 to 7). ***B***: Basaltic subrounded boulder key bed at the base of the Palmasera fan unconformably overlaying a discontinuous beach deposit of carbonate and basaltic pebbles. Note angular boulders tend to occur at the top of the key layers such as the slab (**S**) of log 5; ***O***: local carbonate pebble conglomerate without no basaltic clasts unconformably overlain by the key basaltic boulder layer; **L**: Plio-Pleistocene siliciclastic sandy to pebbly beach deposits; **V:** Plio-Pleistocene basaltic lava cover; **D**: paleosols.; red dot at the base of log 1 and black dot at the base of log 7 indicate location of the obtained post-IRSL luminescence and ^14^C (Coltorti et al. 2010), respectively, numerical dates

Plio-Pleistocene small volcanic vents poured a basaltic cover (10–20 m thick) on the carbonate terrace the Cala Gonone reentrance (Fig. 2a). Part of it forms the substrate of the village almost up to the brink of the conglomerate scarp at Palmasera (**P**) (Figs. 1d, 2). The sedimentary deposits exposed along the coastal scarp, arcuate away from a centrally located feeding stream: the Rio Sos Dorroles codula (sd) that is in turn fed mainly by the Rio Ischirtiore (is) and possibly by streams from the southwestern hills, the main valley toward Dorgali crested at **T** (Figs. 2b, 6a), and from streams of the **S** and **G** fan area (Fig. 2b). The sedimentological characteristics, such as sedimentary structures, indicate these outcrops to be the inner remnant exposures of a relatively large, ancient coastal alluvial fan (Palmasera fan) (Fig. 6). The transversal section has width of 450 m(terminating to the northeast against basalt (**V** of Fig. 6c) and to the southwest against carbonate bedrock (Fig. 2a), maximum thickness of 24–28 m, and a maximum remnant ~ 20 m thick-slice on the scarp-face (Fig. 6c). The longitudinal sedimentary sections along the flanks of the Rio Sos Dorroles codula are not as well-exposed but have similar measurements with a length of 250 m (Fig. 6b).

The Palmasera fan consists of a local southwest outcrop (at the Abba Meica beach), a ‘codula corridor’ including fanglomerate remnants along the flanks and a deep narrow codula (canyon) (Sos Dollore codula) with a torrent, and a northeast portion along the Sos Dorroles beach. In this paper we are going to analyze the better exposed northeastern portion and part of the ‘codula corridor’ (Figs. 2b, 6a).

**Fig. 7:**
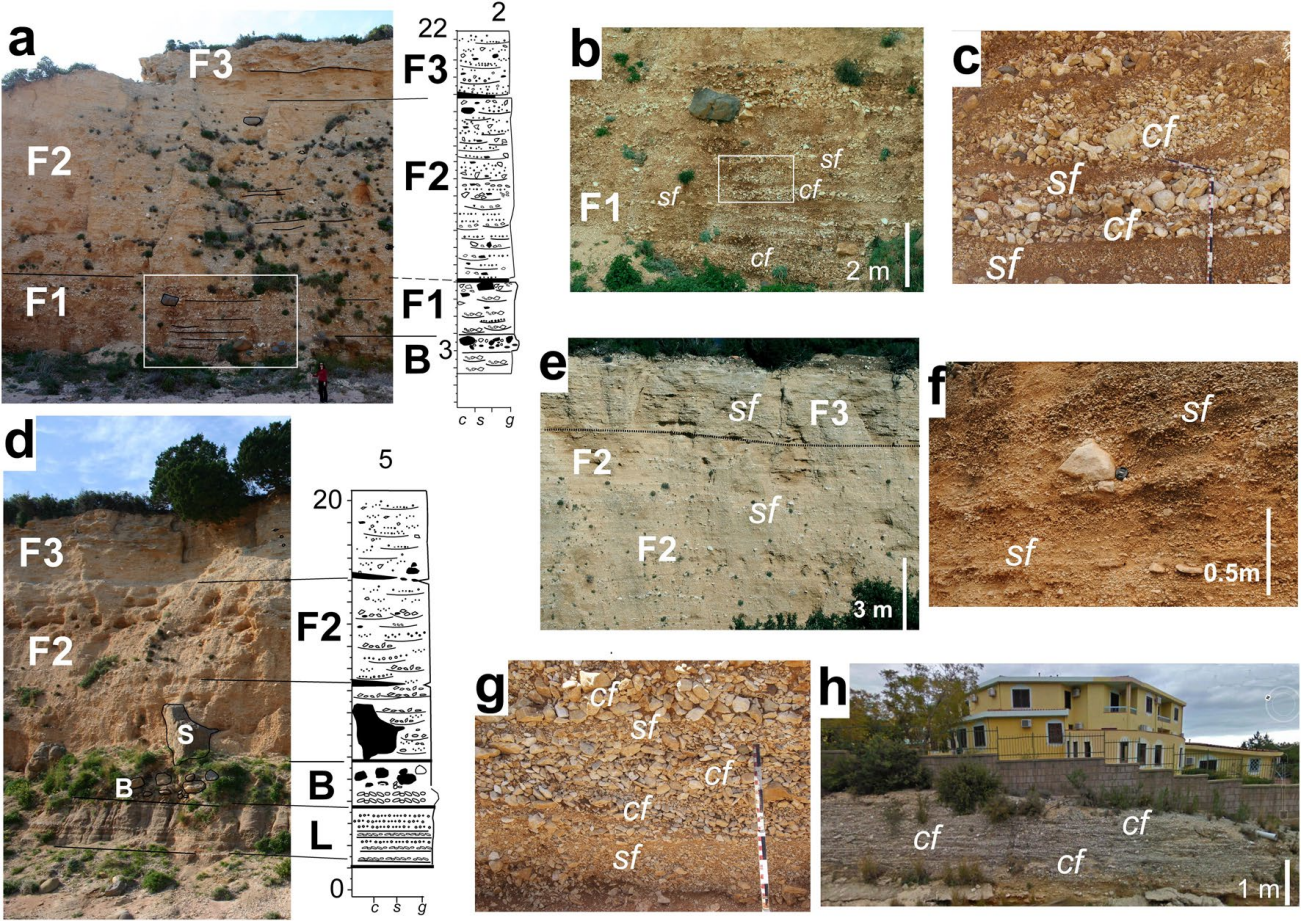
Lithology of the Palmsera fan. **a** View of the alluvial fan at log 2 (Fig. 6c, d) showing the vertical transition between the various units (**F1** to **F3**). The detailed portion of the area with the rectangle is shown in **b**; **b F1 unit:** alternate beds of cobble to coarse pebble conglomerate and of sandy pebble conglomerate made of sheetflood/flashflood (*sf*) and channel flow (*cf*); note the angular basaltic bock most likely rockfalled from the lava crowning the scarp. **c** Detail of the structure of sheetflood/flashflood and channel flow of **F1 unit**; **d**) View of the alluvial fan at log 5 (Fig. 6c, d) showing the vertical transition between **F2** and **F3** and the large basaltic slab **S** at the base; **e** overall fining upward of **F2** deposits and the thinner lenticular layers of **F3** along the left flank of the codula; **f** sheetwash/flashflood events of **F2 unit**; **g** **F3 unit**: alternate of channel (small braided-like channels) and sheetflow at the topmost part of the right flank of the codula; **h** details of conglomeratic sandy layers in a slightly diagonal section of **F3** small outcrop showing a basal poorly sorted flood sandy pebbly deposit overlain by sorted, openwork, sandy gravels channel (*cf*) typical of braided streams. Note the village houses built on top of the conglomerate of Palmasera alluvial fan (for location see the red square in the bottom tight of Fig. 2b). ***B***: Basaltic boulder key bed at the base of the Palamasera fan. **L**: Plio-Pleistocene siliciclastic sandy to pebbly beach deposits

The northeastern coastal alluvial-fan deposits **(F)** generally rest over a basaltic key-bed (**B**) composed primarily of rounded pebbles and large to very large, sub-rounded to sub-angular (at its top) basaltic boulders and few carbonate ones (Figs. 6c, d, 7). The boulders represent rockfalls from the thin basaltic lava cover of the coastal carbonate cliff, in part remolded by wave action in part overlained by preserved angular to subangular ones. This basaltic boulder key bed (**B**) locally overlies unconformably a carbonate and basaltic pebble beach deposits, locally a pebbly conglomerate possibly associated with an older fan (***O,*** Fig. 6c, d). The **B** layer in places overlay unconformably a Late Pliocene–early Pleistocene fine to medium grained silicoclastic, generally sandy to small pebbles, well-rounded, openwork, fine stratified and well-imbricated deposit refereed in part as beach deposit (**L**) (Massari and Dieni,1973) (Fig. 6c, d). The boulder key layer occurs at an elevation of ~ 6 m asl, similar to a well-defined tidal notch occurring all along the coastal carbonate scarps (Antonioli et al. 1999). Other tidal notches occur along these coasts from 7 to 10–12 m above present sea level (asl).

**Fig. 8:**
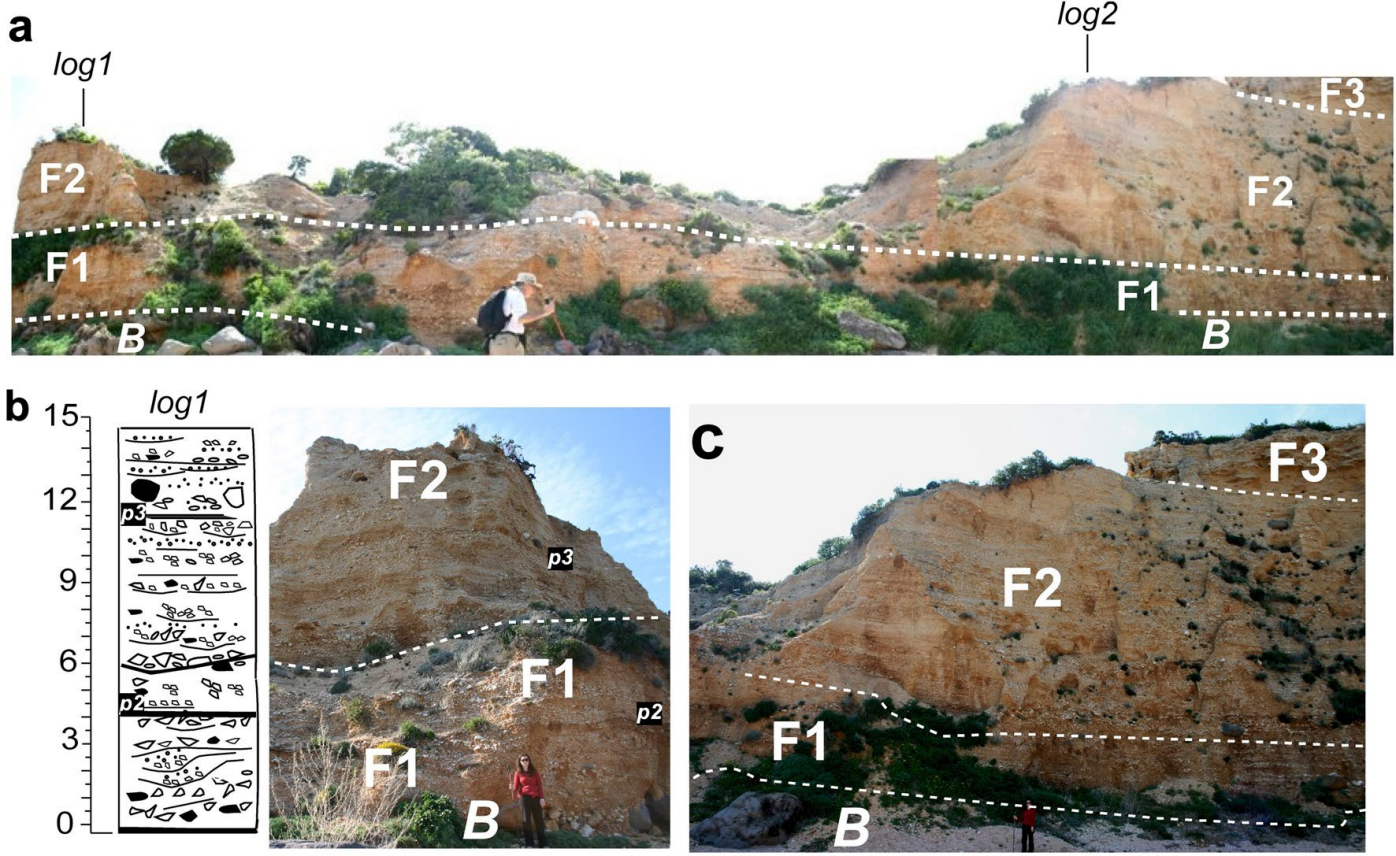
Morphology and remnant sediment section at the mouth (left central side) of the Sos Dorroles codula. **a** Panoramic view of the partially eroded coastal fan at the mouth of the codula between Sect. 1 and 2 (Fig. 6c, d). **b** Log and image of the section showing coarse sedimentation and paleosols (***p2, p3***). **c** Image of Sect. 2 of stratigraphic cross section (Fig. 6c, d) showing also the geomorphologic sequentially irregular downstepwise erosion on the left codula side. **B** = basalt key bed. Person in c: 1.6 m high for scale

The fan deposits **(F)** have similar characteristics throughout, such as poorly cemented sandy conglomerates dominated by carbonate sub-angular pebbles with local cobbles and isolated boulders (Figs. 6, 7, 8). Isolated basalt pebbles, cobbles and few boulders occur sparsely at different horizons. Most layers do not present preferred depositional fabric. Predominant sedimentary structures include definable bedding generally discontinuous and with erosional boundaries (Fig. 6c, d). Along the transversal section subparallel to the coast, slight accumulations of poorly sorted coarser material suggests either channeled or, if domed, debris flow deposits.

Vertical and lateral differences mainly related to changes in grain size and sedimentary structures, and the local presence of poorly developed/preserved, discontinuous paleosols, contributes to a gross subdivision of the **F** deposits into four subunits: **F1, F2, F3** (Figs. 6c, d, 7, 8). Along the transversal section, they are thicker near the codula and thin out to the northeast (Fig. 6).

**Sub-unit F1** is exposed in the lower SW part of the Palmasera section. It is characterized by carbonate deposits with few basaltic boulders and rs (Fig. 7a, b). It shows irregular, lenticular, thin-to-medium-thickness beds of alternating cobble to coarse pebble conglomerate and of sandy pebble conglomerate, and few gravelly (fine pebbles) very coarse-grained sandstone. Some clasts layers are openwork or with very little matrix (Fig. 7c). Carbonate clasts are prevalently sub-prismoidal to sub-discoidal, angular to sub-angular. Conversely, most of the basaltic clasts, except some of the largest boulders, are sub-rounded to well-rounded (Fig. 7b). No definite grain size grading occurs within the beds, some have sub-discoidal clasts showing preferred imbrication (Fig. 7c). The beds are generally sub-horizontal-to-slightly inclined (less than 10°), with sharp basal contact, flat in most layers, slightly concave upward in others indicating shallow cuts-and.

**Sub-unit F2** dominates the central part of the Palmasera section (Fig. 6c, d,). It has a pale yellowish brown coloration similar to F1 and contains a predominance of sandy carbonate fine pebble to granule conglomerates, and pebbly very coarse-grained sandstone beds (Fig. 7a, d, e). The clasts are mainly of carbonate with minor basalt, mostly pebbles to small and few isolated boulders (Fig. 7f). The beds have thin-to-medium-thickness, thinning and lensing out toward the northern termination of the fan body (Fig. 6c, d). They generally have flat, sub-horizontal to slightly concave-up lower boundaries (Fig. 7a, d, e, f). Some shallow, wide cut-and-fill structures occur involving both gravelly layers and, at a smaller scale, sand/granule beds (Fig. 7g). A large slab of basaltic lava (**S**, Fig. 7d) was emplaced in the central lowermost part of the **F2** sequence, indicating rockfall and sliding from an adjacent, still- or re-exposed basaltic scarp.

**Sub-unit F3** is a carbonate sandy and gravelly deposit with no (or rare) basaltic clasts. It is separated from F2 locally by few remnants reddish paleosols and it has a marked difference in structures (Figs. 6d, 7a, d). It has a predominance of thinner lensing beds with more numerous sandy interlayers, fewer cobbles and boulders of carbonate (Fig. 7a, d). The principal structures are shallow cannels, cut-and-fills, cross bedding and laminar structures. Locally has also openwork pebbly gravels alternating with lenticular sandy laminas (Fig. 7g, h).

### Depositional model

**F1** has coarse clasts mainly deposited by powerful floods and some debris flow particularly recorded in residual succession at the codula mouth and nearby sections, and some rockfalls (*rf*) (carbonate and basaltic boulders) from the local coastal scarp (Figs. 6, 7, 8b). Channel flows (*cf*) and sheetfloods/flashfloods (*sf*) predominated in other parts of the unit (Fig. 7b, c).

F2 was not greatly affected by debris flows but by sparse channels and sheetflood/flash flood events (Fig. 7e, f). Paleosols are clearly identifiable in F1 and F2 subunits (Figs. 6d, 7a, d, 8) suggesting time of inactivity. Furthermore, the northeastern part of the Rio Sos Dorroles codula mouth (Figs. 6a, 8) shows a sequential, terminal stepwise erosion of the original sequence.

F3 structures are typical of channel fills with frequent channel migrations and overland deposits (*sf*). In better, smaller exposures along the top, accessible southwest-flank section and near the upstream entrance of the codula, cross-bedding and other small scale structures with various types of vertical and lateral grain size sorting indicate predominance of relatively shallow channels (*cf*) and sheetfloods/flashfloods (*sf*) complex attributable to a braided stream (Fig. 7g, h).

On the whole the Sos Dorroles ( “codula corridor’ (the entrenched codula plus the remnant shoulders fan-deposits: ~ 60 m wide) could have been very active under appropriate critical climatic conditions. It may have acted as the principal route filling and feeding the thickening and expanding coastal Palmasera fan. The sedimentary features of the upper sediments exposed longitudinally along the left (northeast) flank of the entrenched codula have characteristics similarity to the F2 and F3 subunits of the transversal coastal cross section of the fan. Noticeable are structures formed semi parallel or ~ transversally to the main direction of floods (compare Fig. 7a vs e). The last events in this corridor was the entrenchment of the present codula, narrow valley, leaving slices of sediments along the flanks (Fig. 6b: left exposed flank) and in part across the mouth of the ‘codula corridor’ (Figs. 6a, 8). This process of filing and eroding the ‘corridor’ fan deposit may have repeated through glacial and interglacial periods. The downstream Rio Sos Dorroles ‘codula corridor’ and the coastal fan retain traces of the differential erosions from the top of the deposits Log 2 of the stratigraphic section (Fig. 6d) to the base of the present codula stream at sea level in the southwest near the Abba meica (local exposure of the southwest coastal part of the Palmasera fan) (Fig. 8a). The erosional path across the codula mouth indicates discontinuity depending on type of sediments: finer at the top of log 1 (Figs. 6c, 8a, c) to bouldery toward the middle of the codula mouth (Fig. 8b), where a partial column of complete **F1** subunit topped with a partial F2 subunit was preserved. This also illustrates the fact that the sediment deposition and removal from the 'codula corridor’ may have been only partly affected by the variations in sea level. They may have been rather greatly affected by climatic conditions, such extreme rainstorms conditions, and availability of transportable sediments.

## Discussion

### Sediment source

**Fig. 9:**
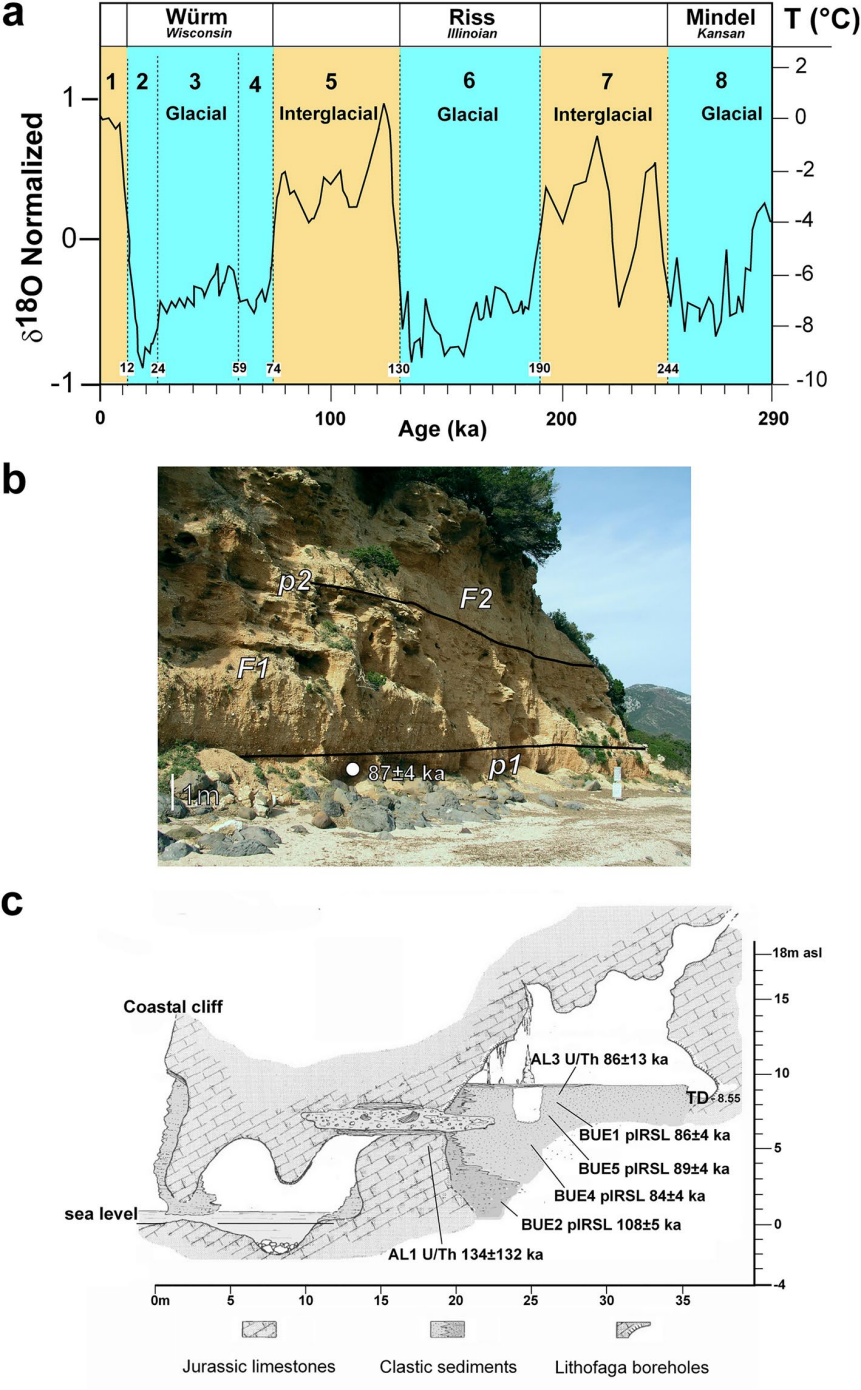
Possible paleoclimatic condition and age of the development of the Palmasera fan. **a** Marine Isotopic Stages (MIS 1 to 8) of the last 290 ka (After Martinson et al. 1987.) Diagram shows the alternate of glacial and interglacial periods occurred in the late Pleistocene. Above are reported the names of the major Alpine (Mindel, Riss and Würm), and North American (Wisconsin, Illinoian and Kansan) glaciations. To the right the normalized value of δ^18^O, to the right the estimated temperature of the last 290 ka (after Kohfeld and Chase 2017). **b** Paleosol ***p1*** sampled for post-IRSL luminescence dating. The sampled section is just south (left of Abba Meica beach). **c** Schematic profile of the aeolian sand filling the Bue Marino cave. With BUE are indicated the luminescence post IRSL ages derived from the aeolian deposits of the cave; with AL the U/Th ages derived from flowstones delimiting the aeolian sand deposits of the cave. TD is the tidal notch referred to MIS5e (After Carobene and Pasini 1982 and Andreucci et al. 2017).

The Pleistocene–Holocene deposits of Cala Gonone have a primary source in the carbonate coastal hills for natural processes and locally, near the top of the saddle between M. Tului and M. Bardia, for human tunnels excavation. Clast formation was favored by thermal, frost and salt-weathering that intensely fractured carbonates (Lanfranchi et al. 2011). Thermal shock, particularly in carbonates (Siegesmund et al. 2000), may have occurred in the Mediterranean area more significantly during warmer interglacial rather than colder and drier periods. Frost shattering, instead may have occurred during cold glacial periods when temperature of Sardinia, for instance, was about 5^0^ to 9 °C lower than the present (Martrat et al. 2004; Pascucci et al. 2014) (Fig. 9a). Although glaciers occurred only in the highest northeast/central area of Sardinia “snow alcoves” are still well-marked in the Cala Gonone lower mountains\hills from where the sediments derived (Fig. 2b). Basaltic megaclasts and boulder- to pebble-sized clasts are secondary constituents of the deposits of Cala Gonone. They derive from rockfalls from the local Plio-Pleistocene basaltic lava of the area. Some clasts have been reworked and partially rounded of by stream/floods and coastal wave flows. Others maintain they native angularity.

### Sediment transport

Similarly, to other world areas, various transport and sedimentation processes can be invoked for the talus cones and alluvial fans of Cala Gonone. Some of them are azonal, such as slump, rock fall, debris falls, debris avalanches, debris flows (cohesive and non-cohesive), hyperconcentrated to high-density flows, and fluid flows (water and wind) (Bertran et al. 1992; Blikra and Nemec 1998; Nemec and Kazanci 1999; García-Ruiz et al. 2001; van Steijn et al. 2002; van Steijn 2011). Most of them require water. Besides generating floods, when in quantity it may increase the weight of the sediment and passively reduces the friction and cohesion between the particles or layers favoring mass movement along slopes (Lorenzini and Mazza 2004). In central-east Sardinia occasional extreme rainfall events have been experienced pouring in a short time, order of few hours or day, large quantity of water, order of 500 mm in few hours (Cossu et al. 2007a, b), leading to debris flows, channel floods and overland sheetfloods (Bodini and Cossu 2008, 2010a, 2010b; Betrò et al. 2008; Hewson et al. 2021). The Stadium and particularly the Gustui fan are dominated in the upper part by remnants of debris flows heavily reworked by sheetfloods/flashflood. Sediments were redistributed over the mid-lower portions of the fans, and may have contributed to the feeding of the coastal alluvial Palmasera fan (**P**). Other sediments transported by streams from the uppermost part to the southwest of the Stadium fan contributed to those funneled through the Rio Sos Dorroles “codula corridor” into the coastal fan (**P**). Additional coarse carbonate and basaltic clasts were added to this fan as rockfall from the open coastal scarp when dislodged by weather and flowing water. The “codula corridor” acted as narrow valley filling up and feeding repeatedly the coastal fan that may have been totally or in part eroded and reformed during upper Pleistocene recurring sea-level oscillations. The Palmasera deposit can, thus, be primarily considered an extension of the inland upper valley talus cones and of lower inland alluvial fans including the Stadium and Gustui ones. The development of such fan would have occurred primarily during period of major climatic changes that led to variation in sea-level and to periods of intense precipitation and floods. Quiescent times or temporarily inactive areas of the fan led to the irregular formation of paleosols. This coastal system experienced deposition mainly during sea regression and significant erosion during sea transgression. Evidence of this has been preserved in the remnant deposits along the coastal scarp at the present sea level.

### Timing

It has not been yet possible to obtain reliable numerical dates directly from the carbonate deposit of the alluvial fans. The only available ages are: those obtained from the basaltic lava (K–Ar 2.11 ± 0.15–2.54 ± 0.11 Ma (upper Pliocene–lowest Pleistocene); the luminescence age of 87 ± 4 ka (late Marine Isotopic Stage—MIS 5) from a putative lowest paleosol (***p1***) of the Palmasera fan in the codula area, and a ^14^C age of ~ 19,365–17,485 a BP (post Late Glacial Maximum, MIS2; Coltorti et al. 2010) from similarly placed putative paleosol (possibly ***p2***) near the northeastern termination of the fan (Fig. 6d). Additional geological information indicating past environmental changes at Cala Gonone are the sediments of the fan themselves as products of the changed climatic conditions leading to their formation, such as mass movements and flood transport. Furthermore, the variations in sea level associated with glacial–interglacial events are indicated by the well-developed tidal notches. All this led to several hypotheses on the formation of the Palamsera fan.

1—The first, oldest by age, hypothesis (Savelli and Pasini 1973; Massari and Dieni 1973) and long followed (Carmignani et al. 2001, 2015) is based on accurate geological studies of the area and the dating of the local basaltic lava flows. It proposes that the fan developed from the Riss glaciation to modern undefined times (Fig. 9a).

A difficulty with this hypothesis is that the relatively small Palmasera fan, albeit supplied by a comparative large drainage area, had its inner parts (the present residual exposed slice) persisting or reforming trough two glacial and interglacial periods.

2—A second hypothesis proposes the Palmasera fan to have formed or reformed after the last glaciation during the relatively short time spanning from the post late Glacial Maximum (MIS 2) and the recent interglacial stage (MIS1) (Fig. 9a), experiencing various strong climatic changes (Coltorti et al. 2010).

One difficulty with this hypothesis is that it is based on a single ^14^C date from a putative paleosol at an elevation reachable by strong waves that my lead to contamination.

3—A third pacifying hypothesis suggests that the formation of the Palmasera fan started after the last interglacial period during the following cooling stages (Fig. 9a). The hypothesis is based on a single potassium luminescence age from a putative paleosol horizon reachable by potentially storm waves (Fig. 9b), and on numerical dates of the siliciclastic deposits inside the proximal, coastal Bue Marino cave deposits (Figs. 3b, 9c). This hypothesis is in agreement with numerous last-interglacial systems around Sardinia including our reconstructions made in northwest Sardinia such as Alghero (Andreucci et al. 2010) and Argentiera (Andreucci et al. 2014; Pascucci et al. 2014).

If Cala Gonone alluvial fan developed mostly during last interglacial–glacial time, that is from about MIS5 to about MIS4 and MIS3 (Marine Isotope Stages) (Fig. 9a), it is possible to hypothesize that any pre-existent fanglomerates may have been mostly removed during the higher sea levels (around maximum MIS5 interglacial). This could have been followed by major buildup of the new fans when the glacial-conditions advanced, hence sea-level retreat, ~ MIS4, ~ MIS5 (Fig. 9a). We are now at a rising sea level stage as the glaciers melt and retreat, and is expected the processes to continue caused in part by the anthropogenic deterioration of the atmosphere continuing the warm-up (Fig. 9a).

Independently of the accuracy of determining the date of initiation or reformation of the fan, at some time the “codula-corridor” was filled and the fan developed extending seaward. This occurred primarily by availability of much sediment and environmental factors capable to transport it. The sea level may have had only a secondary influence in the building of the fan and filling of the codula. Its rising level, however, had and still has a primary influence in eroding most of the Palmasera fan.

## Hazards/risks

Great effort is made to globally recognize the hazards and the risks of alluvial fans and possible remediation if necessary. Much information on the occurrence of disasters is available and good guidelines have been written for detailed surveys of the landscape, taking also account of climatic and geological/geomorphological conditions (such as structure, stratigraphy, sedimentology, numerical dating procedure and geotechnical characteristic of the bedrock and sedimentary deposits). Guidelines and specifications for flood hazard and mapping may be found in papers, books and reports (FEMA 2000, 2021; Lancaster et al. 2015; Da Silva Nascimento and Alencar 2016). Lancaster et al. (2015, p.1) stressed the geological approach to “*identify the general distribution of alluvial fans, the relative age of alluvial deposits, and the relative likelihood of alluvial fan flooding*”. Extending this approach to detailed sedimentological analysis, possibly enhanced by difficult to obtain numerical dating of the deposits, would allow precise prediction of revival of the dormant older fans under extreme climatic variations. Indeed, we are now experiencing increasingly frequent worldwide short intense rainstorms readily triggering mass sediment movement and extreme floods associated with persistent warming and more variable climatic conditions.

**Fig. 10:**
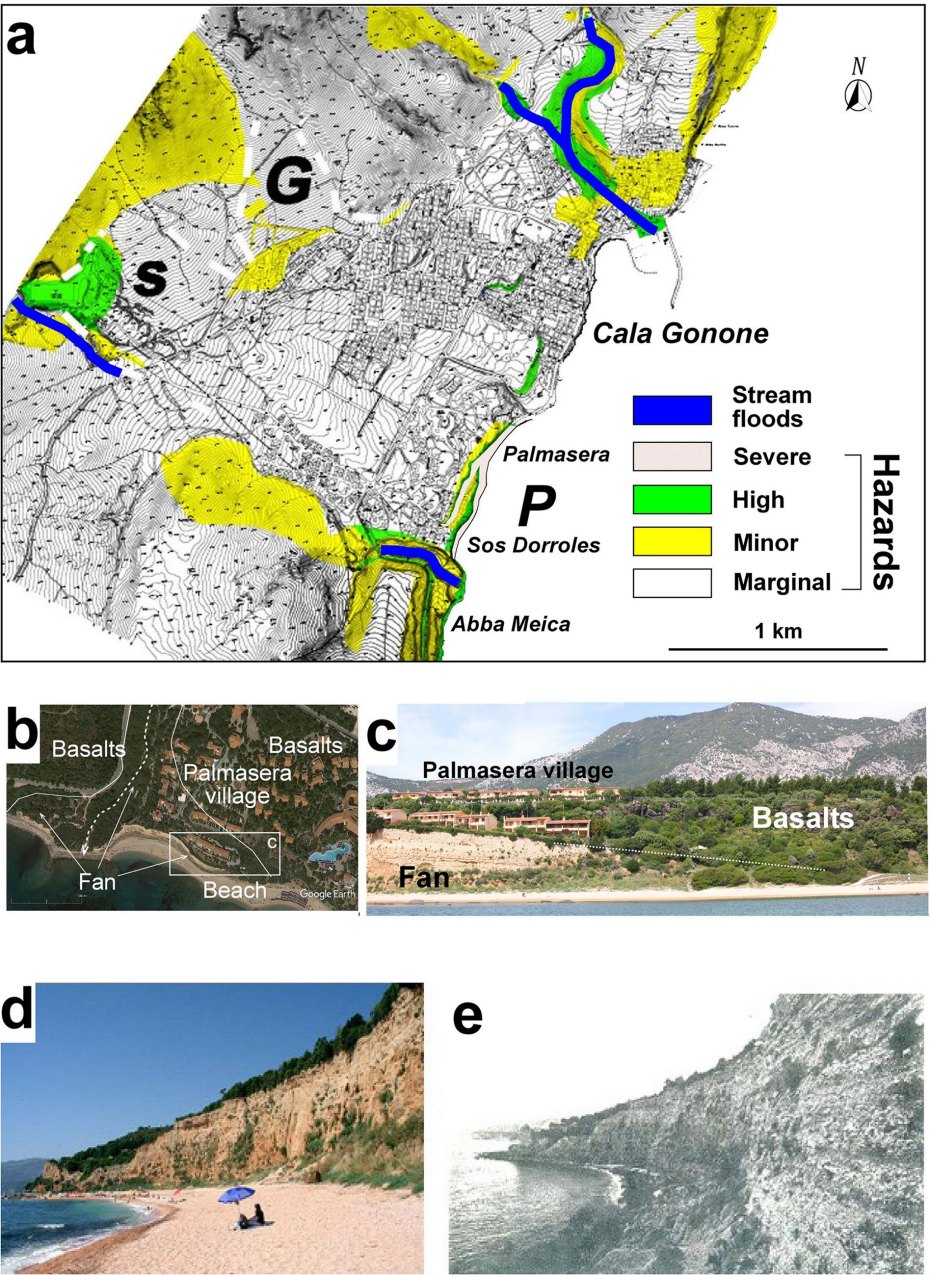
Coastal hazards in the Cala Gonone area. **a** Map of the principal slumps and floods (blue) hazards of the Cala Gonone area (modified from the map of the 2010 report on Piano Urbanistico Comunale of Dorgali Municipality (PUC), maps are available at: https://www.comune.dorgali.nu.it/area-tecnica/attivita-e-servizi/piano-di-protezione-civile.html. Stadium (***S***), Gustui (***G***) and (***P***) Palmasera alluvial fans). **b, c** Satellite and ground views of the northeastern terminal part of the Palmasera alluvial fan with village houses dangerously built near the brink of coastal. **d** Modern Palmasera beach replenished with carbonate and granitic sand in mid 1990s, which in part protects the remnant Palmasera cliff from erosion. **e** Early 1990s Palmasera pre-replenishment beach and conglomerate cliff undergoing significant erosion. Note the persistent rockfall hazard (after Pranzini and Mania 1996)

Few considerations can be made on the information obtained from geological, geomorphological and dating of the Cala Gonone alluvial Stadium and Gustui. Their deposits attest to powerful mass wasting, debris flows and flashflood events from the mountain creeks and erosion by storm waves along the coast. Such hazards still exist. Indeed streams such as those now flowing at Cala Fuili and Luna (Fig. 3b) are experiencing occasional strong floods remolding the coasts (Cossu et al. 2007a, b). Furthermore, as reported in reports on the landscape of the area prepared for the municipality of Dorgali, floods hazards and risks have been single out for the Rio Irvieri and Rio Ischirtiore–Sos Dorroles codula flowing, respectively, at the northeast and southwest limit of the village (Fig. 10a) (https://www.comune.dorgali.nu.it/area-tecnica/attivita-e-servizi/piano-di-protezione-civile.html (in Italian). These potential flooding streams constitute significant hydrologic hazards but not much potential risk, considering they flow in limited populated areas or have recurrences of more than 10 ^+^ yr, allowing timely remediations. More ominous hazards and ensuing risks exist from possible sediment ridden debris flows and sheetfloods\flashfloods flowing from the hills and reworking **S** and **G** fans through the village (Figs. 2b, 7h). They are likely to reoccur in future in unpredictable time and intensity. Their happening is tied to sudden, short duration, yet unpredictable mega-pluvial events as already experienced in Sardinia and possibly increasingly in a future hotter climate due to continuous warming, recurrent extreme floods, heatwaves, droughts and storms (https://www.severe- weather.eu/tag/sardinia). Moreover, cyclones such as the recent Cleopatra (Niedda et al. 2015) and high intensity sudden flashfloods are becoming normally occurring events (Amponsah et al. 2018).

Such damaging processes, however, may have centennial or millennial recurrence tied to the cyclicity of glacial–interglacial events (Fig. 9a). Nevertheless, considering that warming climate is still intensifying, attentive warnings and remediation should be continued. An example of a possible great man-made risk is to misjudge the power of natural processes such as those comminuting carbonate rocks forming considerable new sediments available for transport also in worm intergacials. This is the case of the coastal carbonate hills of Cala Gonone. They have been intensely tectonically fractured both at the macroscopic and microscopic scale and have a range of intercrystalline porosity (Lanfranchi et al. 2011). Intense solution has developed surficial structures, numerous karst caves (De Waele 2004) as well as enlarged fracture porosity. The rock-temperature variation (insolation variations) may lead to thermal stress, fatigue and thermal shock particularly in carbonates (Siegesmund et al. 2000). Under these conditions the rock may start to deteriorate, microfractures may enlarge, and the rock may exfoliate and comminution occurs. Salt weathering is another process that leads to comminution of rocks as indicated by the deterioration of ancient monuments and observed in various environments including hot and cold desert areas and, routinely, in coastal areas (Goudie et al. 1997; Goudie, 2000; Hall et al. 2002; Hartley et al. 2005). As marine water liquid or moist transported by winds and fogs penetrates into the rock pores, it carries dissolved salts that can precipitate as drying occurs because of evaporation or cooling. Rock weathering thus may act through crystallization pressure and thermal expansion, osmotic pressure and chemical weathering. Repeated cycles of crystallization and solution of soluble salts within the pores lead to damage and comminution of the rocks (Coussy, 2006; Ruiz-Agudo et al. 2007). Much sediment can be generates and added to what can be reworked from hat is already in the alluvial fans can be catastrophically transported over parts of the village. Monitor of the amount of sediments in the drainage area, perhaps preparing sluice to direct any debris flow or debris flood could be considered. Unfortunately the incipient aridification indicates the upper fan areas may not be suitable for the plantation of forest barriers.

Furthermore, Cala Gonone, such as many other Earth locations, is also affected by problems associated with ongoing sea-level rise (Antonioli et al. 1999). The main effect of this is the erosion of coastal beaches and collapse of the remnant sedimentary slices of the ancient coastal fans such as the one in the frontal Palmasera scarp (Arba et al. 2002). There modern village habitations have been built near the brink of the coastal fan scarp on the edge of the basaltic lava (Figs. 6a, 7h, 10b, c). The houses stat to show structural weaknesses and they are likely to experience increasing damage as the sedimentary conglomerates will continue to be removed and eventually the newly most exposed basalts may collapse as it happened long time ago (slab shown in Fig. 7d). This process has been temporarily remediated by an extensive replenishment of the beaches Abba meica and Sos Dorroles in late 1990s in front of the residual Palmasera fan (Figs. 6a, 10d, e) (Pranzini and Mania 1996; Arba et al. 2002). The replenishment has also added a great touristic beach attraction and significantly reduced wave erosion risk of the coastal cliff of the Palmasera village. The effort should and will be made to maintain also the subaqueous wave breakers remediation to keep beach replenishment in good state, and continue to reduce the potential rock fall and slumps risk from the fan scarp (Fig. 10d).

## Conclusions

Pleistocene to Recent sedimentary deposits like those associated with rocky coasts of the Gulf of Orosei have a scarce to nil probability of preservation in the geological record. Nevertheless their sedimentological studies have still intrinsic scientific and practical values emphasizing the complexity of the sedimentary facies (aspects) that have developed reflecting the influence of multiple processes in different parts of the systems under different climatic conditions. There is a tendency to create manageable, simple, useful models of systems for colluvial and alluvial fans. They are, however, complex features of our landscape, which need to be understood also sedimentologically to contribute effectively to landuse, safe-keeping and sensible future development planning. The Cala Gonone region is tucked in the eastern side of steep mountains/high hills. The modern village persisted quasi-isolated from the rest of Sardinia for most of the initial part of the twentieth century. In the last half of the last Century, good roads and modern tunnels were realized and strong touristic development occurred. This led to the rapid expansion of the village that, needing new land, necessarily constructing the new buildings on the wide alluvial fan deposits on to the marine rentrance. Considering the increasing climatic deterioration with associated anomalous strong rainstorms, floods and slumps, problems may be envisaged to occur. To this purpose the Municipality of Dorgali has promoted a detailed landscape study of the region to optimize resources, developments, and prevention of major disruptions.

The village of Cala Gonone now covers 1/3 of the lower part of two major alluvial fans (Stadium (**S**) and Gstui (**G**)) and it is still enlarging. Although now dormant, the two fans are likely to experience floods cutting unexpectedly through parts of Cala Gonone. In addition, storm waves may undermine the residual coastal fan (Palmasera (**P**)) and eventually the solid rock scarp.

The sedimentological analysis of the Cala Gonone alluvial fans reported here deals with hazards and risks for the enlarging village. It provides information on processes, past results, and may give an idea of the generic timing of the occurred events, but in the carbonate deposits cannot yet give numeric dates. It seems, however, reasonable hypothesis those sediments were mostly generated in part through cold climate freeze-and-thaw cycles and coastal warm-climate weathering t and their transport occurred through sediment-gravity flows such debris flows and flash floods triggered by major rainstorms. Such intense rainy events and movements of sediments are occurring today as well in.

Cala Gonone is a small, typical, recently enlarging site waiting for something to happen, perhaps in hundred to thousand or more years depending on future climatic changes. The probability of debris flow sand floods exists but the risk is relatively low because of the long, order of 10–100 + year of recurrence. No remediation has been apparently applied for inland problems, even a forested endeavour may be difficult on those dry, arid terrains. The hazard associate to the erosion of the residual coastal fan deposits exists but the risk is affecting a small part of the village and the guests using the beach. The erosion of the remaining slice of the ancient fan will continue but the re-nourishment of the beach is a good temporary protection. Continuous alert and some local additional remedial work would be advisable.
